# Deciphering GRINA/Lifeguard1: Nuclear Location, Ca^2+^ Homeostasis and Vesicle Transport

**DOI:** 10.3390/ijms20164005

**Published:** 2019-08-16

**Authors:** Víctor Jiménez-González, Elena Ogalla-García, Meritxell García-Quintanilla, Albert García-Quintanilla

**Affiliations:** 1 Department of Pharmacology, School of Pharmacy, University of Seville, 41012 Seville, Spain; 2 Institute of Biomedicine of Seville (IBiS), University Hospital Virgen del Rocío/CSIC/University of Seville, 41013 Seville, Spain; 3 Department of Biochemistry and Molecular Biology, School of Pharmacy, University of Seville, 41012 Seville, Spain

**Keywords:** GRINA/TMBIM3/LFG1, calcium homeostasis, endoplasmic reticulum stress, endosome-to-Golgi retrieval, vesicle, alternative splicing, sterol, nucleolus, Proline-rich domain, interactome

## Abstract

The Glutamate Receptor Ionotropic NMDA-Associated Protein 1 (GRINA) belongs to the Lifeguard family and is involved in calcium homeostasis, which governs key processes, such as cell survival or the release of neurotransmitters. GRINA is mainly associated with membranes of the endoplasmic reticulum, Golgi, endosome, and the cell surface, but its presence in the nucleus has not been explained yet. Here we dissect, with the help of different software tools, the potential roles of GRINA in the cell and how they may be altered in diseases, such as schizophrenia or celiac disease. We describe for the first time that the cytoplasmic N-terminal half of GRINA (which spans a Proline-rich domain) contains a potential DNA-binding sequence, in addition to cleavage target sites and probable PY-nuclear localization sequences, that may enable it to be released from the rest of the protein and enter the nucleus under suitable conditions, where it could participate in the transcription, alternative splicing, and mRNA export of a subset of genes likely involved in lipid and sterol synthesis, ribosome biogenesis, or cell cycle progression. To support these findings, we include additional evidence based on an exhaustive review of the literature and our preliminary data of the protein–protein interaction network of GRINA.

## 1. Introduction

In this paper, we review the existing literature about Glutamate Receptor Ionotropic NMDA-Associated Protein 1 (GRINA), and combine it with different software tools to analyze in depth the domains and motifs present in the protein, as well as transcription factors involved in its expression, with the aim of deciphering potential functions, circumstances and mechanisms relevant to GRINA, with an emphasis in mental and neurodegenerative disorders. Lastly, we present preliminary data about potential ligands of GRINA that reinforce the concepts exposed.

## 2. The Controversial Discovery of GRINA and Its Alternative Names

Several decades ago, the scientific community made an intense effort to clone the NMDA (*N*-methyl-d-aspartate) receptor. This research was justified by the fundamental role of such ionotropic glutamate receptors in neurobiology. Finally, in 1991, two teams claimed that they were able to clone it. The receptor published by Nakanishi’s group [1] showed sequence homology with the AMPA and kainite receptors (both also ionotropic glutamate receptors), and, like them, it had four transmembrane regions. However, the glutamate-binding protein subunit cloned by Michaelis et al. [2], later known as GRINA (Glutamate Receptor, Ionotropic, NMDA-Associated Protein 1) or NMDARA1, contained seven transmembrane α-helices and lacked any homology with other glutamate receptors, raising questions about whether it could have other functions [3]. To measure its ligand activity, rat GRINA was cloned in *Escherichia coli* and purified from the bacterial extracts by affinity chromatography on glutamate-treated columns, displaying an estimated dissociation constant of 263 nM for glutamate [2]. According to the authors, it was part of an NMDA receptor-like complex formed by 4 subunits (the glutamate-binding protein corresponding to GRINA, the glycine-binding protein, the carboxypiperazinylphosphonate-binding protein, and the phencyclidine-binding protein) [4,5]. To evaluate the formation of ion channels, they reconstituted the protein complex (previously isolated from rat brain synaptic vesicles) into liposomes and measured their activity using voltage clamp techniques following their fusion with planar lipid bilayer membranes. This way, they determined that the receptor was dependent on the presence of glycine for optimal activation by glutamate and NMDA, with a predominant conductance state of 47 pS and a secondary one of 23 pS [6]. When the mRNA levels of GRINA were examined by in situ hybridization in the rat brain, they showed a similar expression pattern to the NMDAR1 receptor, except for the hypothalamus, where GRINA was not expressed, and the parafascicular nucleus of the thalamus, where NMDAR1 was not present [7]. After immunocytochemistry with antibodies developed by their own team, staining was observed in the cell body and dendrites of pyramidal neurons from the hippocampus and layers II, III, and V/VI of the cerebral cortex, as well as granule cells from the dentate gyrus, cerebellar cortex, and the olfactory bulb, also including the mitral cells of the olfactory bulb [4,8]. However, unlike in situ hybridization, no staining was observed in Purkinje cells [9].

**Figure 1:**
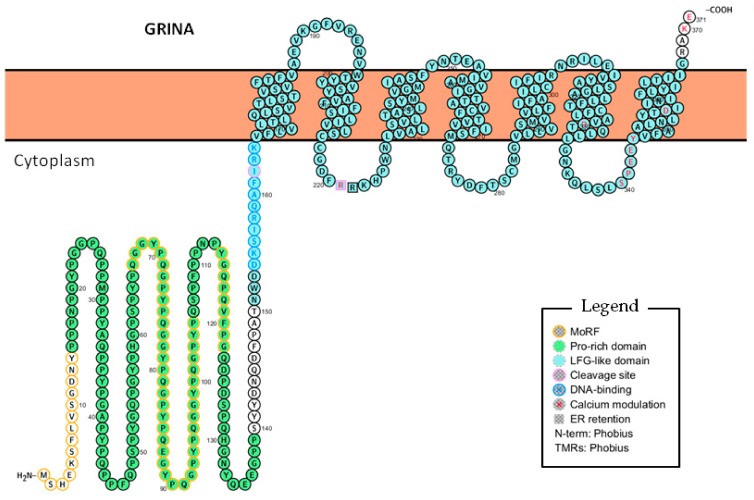
General representation of human Glutamate Receptor Ionotropic NMDA-Associated Protein 1 (GRINA) highlighting its domains and the most relevant features. The graph was generated using Protter v1.0 [17].

Despite the cDNA cloned by Michaelis et al. in 1991, matched with *GRINA* (except for a few point mutations), the initial amino-acid sequence inferred by them was partly incorrect [2], probably hampering that the results could be reproduced by other teams. In 2011, Nielsen et al. proposed a revised initiation codon and open reading frame (ORF) for the rat GRINA [10], resulting in a 347 amino acid protein (instead of 516 residues) with a predicted molecular weight of 38.5 kDa (instead of 57.0 kDa). The resulting protein, like the original candidate described by Michaelis et al., showed a central part containing the seven transmembrane α-helices, but diverged in the first 82 N-terminal residues and the last three at the C-terminal side, plus all the extra ones after the stop codon (now absent). This was due to the use of a wrong starting AUG codon and the presence of point insertions/deletions mainly caused by repeated nucleotides (typical of low quality sequences) that changed the frameshift and skipped the stop codon. When the expression of GRINA was measured by Northern blot in different murine tissues, it resulted in a broad expression pattern, with high levels in the hippocampus but also in the cerebellum, basal ganglia, cortex, and hindbrain, as well as other organs like the kidneys and testes [10]. These mRNA expression results were compatible with the ones previously obtained by Michaelis’ team, due to the sequences used for the probes. However, the protein now displayed homology with a superfamily of proteins present in eukaryotes, prokaryotes, and some orthopoxviruses that contain the cytoprotective BAX inhibitor (BI)-1 motif. Therefore, an alternative name TMBIM3 (Transmembrane BAX Inhibitor Motif 3) was proposed. In humans, the TMBIM superfamily includes six members: TMBIM1 (RECS1), TMBIM2 (FAIM2), TMBIM3 (GRINA), TMBIM4 (GAAP), TMBIM5 (GHITM), and TMBIM6 (BI-1) [11]. The members of this superfamily are involved in programmed cell death and survival and are able to reduce endoplasmic reticulum (ER) Ca^2+^ content [12,13]. TMBIM1 and TMBIM2 participate in inhibiting extrinsic apoptosis. On the other hand, TMBIM3/GRINA and TMBIM6 inhibit intrinsic apoptosis, regulating Ca^2+^ release trough IP_3_-activated Ca^2+^ channels (IP_3_Rs). TMBIM4 also regulates IP_3_Rs and is able to inhibit both inhibit intrinsic and extrinsic programmed cell death. TMBIM5 is the only member expressed in the mitochondria, and the only one not able to reduce basal Ca^2+^ concentration in the cytosol [11]. They all have seven transmembrane regions, but unlike G protein-coupled receptors, their N-terminus is cytoplasmic instead of extracellular, and GRINA is the member with the longest cytoplasmic tail (Figure 1). Subsequent phylogenetic analysis in eukaryotes showed that they could be further divided into the BI family that included BI-1 and GHITM, and the Lifeguard (LFG) family [14,15], which gave rise to a new recommended nomenclature: LFG1 (GRINA), LFG2 (FAIM2), LFG3 (RECS1), LFG4 (GAAP), and the testis-specific human pseudogene LFG5 (TMBIM7P) [16]. All but LFG4 originated via duplications of a LFG1-like precursor [14]. However, this division is not functional, and only occurs from a phylogenetic point of view.

Despite the validity of the in situ hybridization results obtained by Michaelis et al. for the mRNA expression of GRINA, it remains unanswered if the glutamate-binding protein described by them is the same as TMBIM3/LFG1, a chimera, or a completely different protein. To solve this question, a sequencing of their protein would be necessary, or at least to confirm its detection using commercially available anti-GRINA antibodies, in addition to their own developed antibodies. For this reason, this review focuses only on the human GRINA/TMBIM3/LFG1 equivalents to the protein deduced by Nielsen et al. in rodents.

## 3. The Gene Coding for GRINA

Human *GRINA* is located at the chromosome region 8q24.3, near the subtelomere [18]. *GRINA* codes for a protein of 371 amino acids, with an estimated molecular weight of 41.2 kDa. The gene contains seven exons. Six of them span an ORF, and, strikingly, the first two coincide exactly with the N-terminal region of GRINA (www.ncbi.nlm.nih.gov/gene/2907). The two reference transcript variants currently described (NCBI accession numbers NM_000837.1 and NM001009184.1) only differ in their untranslated regions (UTRs). In addition, other UTRs have been reported in prostate adenocarcinoma (NCBI accession BC041788) and kidney (NCBI accession AK127640). An alternative splice variant lacking the sequence PPPNPGYPGGPQPPMPPYAQ (fragment 15–34) has been found in the Alzheimer cortex (NCBI accession AK294127), but its relevance is still unknown. Moreover, alternative splicing of GRINA has been reported in horn cancer, a very common type of squamous cell carcinoma, in Zebu cattle [19].

## 4. Deciphering the Functions of GRINA Through Its Domains and Motifs

In order to advance in the characterization of the potential functions and mechanisms of GRINA, we decided to analyze the presence of domains and motifs.

Motifs are conserved amino acid patterns of biological significance that usually mediate a common function. We searched for the presence of linear motifs using the eukaryotic linear motif (ELM) resource [20] online. The conserved results are shown in Table 1.

Domains are defined as independent folding units that usually carry a specific function within the protein. When analyzed with the NCBI’s Conserved Domains Database (CDD) [21], GRINA displayed two major domains: a Pro-rich domain within the N-terminal tail and an LFG-like domain, belonging to the BI-1-like superfamily, across the transmembrane region (see Figure 1). The main findings are discussed below.

### 4.1. The Lifeguard Domain of GRINA

The LFG domain gives its name to LFG1, GRINA’s alternatively recommended name. As commented before, mammalian members of the BI-like superfamily include transmembrane proteins related to cell death and survival. All TMBIM members, and therefore Lifeguard members with an ER location, mediate ER Ca^2+^ homeostasis and suppress intrinsic apoptosis pathways by reducing cytosolic Ca^2+^ concentration upon ER stress [11]. Cytosolic Ca^2+^ concentration is maintained low at around 100 nM, in contrast to ER and Golgi apparatus, where it is 3–5 thousand fold higher than the cytoplasm. Under prolonged stress conditions, Ca^2+^ can exit from the ER, enter the mitochondria, and trigger cell death [12].

Three models have been proposed to explain how TMBIM proteins arbitrate Ca^2+^ homeostasis. The first model is based on the presence of a Lys-rich motif at the C-terminus of TMBIM6 (KE 370–371 for GRINA) (Figure 1). This model has a certain similarity to the Na^+^ channel [12] and may be involved in the Ca^2+^/H^+^ antiporter, but also in regulating cell adhesion [14].

**Figure 2:**
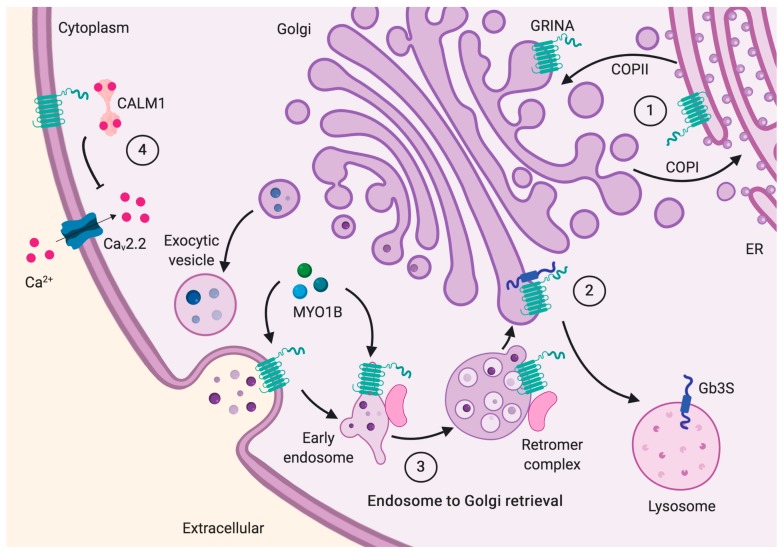
The role of GRINA in vesicle traffic and sorting. (1) GRINA may translocate from the endoplasmic reticulum (ER) to the Golgi using coat protein complex II (COPII) with the assistance of ALG2 in response to a Ca^2+^ increase or have the exposed di-Arg motif recognized by COPI for ER retrieval. (2) GRINA may interact with Gb3 synthase (Gb3S), redirecting it to the lysosome. (3) GRINA is required for Endosome-to-Golgi retrieval where it may associate with other proteins like MYO1B. (4) GRINA has been observed also in the plasma membrane where interacts with Ca_V_2.2 channels (abundant at the presynaptic neuronal terminals) to participate, in collaboration with Calmodulin (CALM1) or G-protein β_γ_ subunits, in the regulation of the cellular Ca^2+^ influx and the release of neurotransmitters.

The second mechanism is based on the high homology of the LFG domain with the bacterial TMBIM protein YetJ from *Bacillus subtilis* [22]. According to this model, at pH 8, the channel is closed, open at pH 6, and both structures coexist in equilibrium at pH 7. When closed, Asp171 from transmembrane 6 (TM6) and Asp195 from TM7 form an H-bond, and Asp171 is negatively charged, forming an H-bonded-salt bridge with Arg60 from the second intermembrane region. However, at pH 6, Asp171 protonates and disrupts its interaction with Arg60, opening the Ca^2+^ pore. These residues are conserved among the TMBIM family, and for GRINA, they correspond to Asp327 (Asp171), Asp354 (Asp195), and Arg221 (Arg60). It is relevant to mention that Arg221 is part of the di-Arg motif within the second intermembrane region that faces the cytosolic side and serves as an ER-retrieval and retention motif for ER localization, guaranteeing the correct folding and assembly of multimeric proteins [23]. Thereby, when the di-Arg motif is not exposed, either by correct folding, proper complex assembly, or masking by a PDZ domain-containing protein, GRINA can escape the ER and travel to the Golgi or the cell surface. However, if exposed, it can be recognized by the vesicle coat protein complex I (COPI) for ER retrieval. Interestingly, the di-Arg motif is also present in other ionotropic and metabotropic glutamate receptors [24,25], and, in the case of GRINA, it overlaps with an *N*-Arg dibasic convertase cleavage site (R^RK, position 221–223) (see Table 1; Figure 1 and Figure 2), which may contribute to the fine control of its location.

The SPEEY motif (position 340–344) (Figure 1) is also central within the channel pore and important for cell adhesion and migratory functions [15]. Ser340 may become phosphorylated either by the anti-apoptotic casein kinase 2 (CK2) or MAPKs, and once phosphorylated, be recognized by the peptidyl-prolyl cis/trans isomerase Pin1, which is involved in processes such as cellular stress response or neuronal function. This Pro341 isomerization may well provide a conformational regulation mechanism for GRINA.

A binding motif for TRAF2 (sequence SPEE, position 340–343) is present in the sixth intermembrane region of GRINA, facing the same side of the N-terminal domain. TRAF2 (TNF receptor-associated factor 2) is a cytosolic protein that is recruited to membrane associated receptors and has a strong E3 ubiquitin ligase activity in the presence of sphingosine-1-phosphate [26], where it is able to interact with Inhibitors of Apoptosis (IAPs) to serve as an anti-apoptotic signal, by regulating the activation of the transcription factors NF-κB and Ap-1. Curiously, the only ubiquitination site predicted for GRINA using the UbPred software [27] is located in the N-terminal Lys5 residue, facing the same side as the TRAF2 binding motif.

In the third mechanism, GRINA would function as a sensitizer of Ca^2+^ channels, such as the IP_3_Rs under ER stress [28], or the N-type voltage-gated Ca_V_2.2 channels in the plasma membrane [29]. The ability of GRINA to interact with IP_3_Rs to regulate ER stress also occurs with other pro-survival proteins like TMBIM6, Bcl-2, and Bcl-XL [12]. In particular, GRINA is able to interact with IP_3_R3 and IP_3_R1 [28], as IP_3_R1 is highly enriched in the cerebellum and the brain, including hippocampal neurons [30]. In addition, the interaction of GRINA with TMBIM6 has a synergistic effect on IP_3_Rs and Ca^2+^ regulation [28]. On the other hand, the interaction of GRINA with the domain IV of the Ca_V_2.2 α1 subunit has modulatory effects comparable to G-protein β_γ_ subunits [29].

The C-terminal side of GRINA, but not the full GRINA containing the N-terminal cytoplasmic tail, is able to reduce the presence of Globotriaosyceramide (Gb3) on the cell surface (thus conferring resistance to Shiga toxin) by redirecting the Gb3 synthase to the lysosomes and promoting its degradation (Figure 2). Physical interaction has been demonstrated by the immunoprecipitation of the C-terminal part of GRINA and Gb3 synthase. All other members of the TMBIM family displayed a similar effect [31].

### 4.2. The Pro-Rich Domain of GRINA

Around one third of eukaryotic proteins contain intrinsically disordered regions (IDRs) [32]. This implies that these proteins do not have a single stable structure under native conditions but, instead, can adopt different conformations with unique functional capabilities not achievable by rigid structured regions [33].

The flexibility of IDRs allows proteins to bind a broad range of ligands according to the varying physiological needs. However, these unions are more transient and weaker than those occurring in folded proteins due to their lower entropy upon binding [34].

IDRs are present in transcription factors [33], in one-half of RNA chaperones, and in one-third of protein chaperones [34]. They participate in key processes, such as the assembly of complexes (like the ribosomes), secretion, biomineralization, alternative splicing, and post-translational modifications relevant in regulation and signaling [34]. Indeed, IDRs tend to undergo post-translational modifications and are enriched for sites (Ser, Thr, Tyr) that can be phosphorylated by kinases [32,34]. Such phosphorylation sites usually occur within Pro-rich regions [35]. Remarkably, IDRs lean to bear Pro-rich sequences that confer them propensity for the polyproline II (PPII) conformation [35] and aromatic residues that contribute to the orientation of the ligand during the interaction [36]. Several motifs recognized by SH3 domains are present along the N-terminal half (Table 1). This is logical, since SH3 domains recognize PPII helices and are involved in organelle assembly, membrane traffic, cytoskeleton organization, or signal transduction.

After using the D^2^P^2^ database of disordered protein predictions [37], more than 75% of the predictors agreed on the presence of an IDR at the N-terminal half of GRINA spanning the first 145 residues. Just four amino acids (Pro, Gly, Gln, Tyr), mainly located within the 11–148 positions, represented two thirds of the N-terminal region. Proline is the most abundant of all of them, with 47 residues (49 in the entire protein) and gives its name to the Pro-rich domain. The majority of Pro residues are distributed in six PP pairs and seven PYP triads, while 70% of Tyr residues are evenly spread as YX{4}Y.

When analyzed with the PhosphoMotif Finder software [38] 12 potential anaplastic lymphoma kinase (ALK) binding motifs (YX{4}Y) [39] were found within the IDR, seven of them regularly distributed. It is noteworthy to mention that ALK plays an important role in the development of the brain. Tyr is the more abundant phosphorylation-competent residue at the N-terminal region (20 times), followed by Ser (9 times), and only one Thr, representing together almost 20% of the N-terminal residues.

Naaby-Hansen et al. [40] demonstrated that a protein can gain a Ca^2+^ binding capacity through Tyr phosphorylation. This process could also be relevant for GRINA, allowing high concentrations of Ca^2+^, while preventing the precipitation (calcification) of the corresponding salt [41]. The analysis with IonCom [42] reported that GRINA is able to bind to Zn^2+^, Ca^2+^, and Mg^2+^ cations.

**Figure 3:**
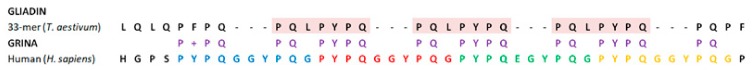
Homology between the 33-mer gliadin peptide and human GRINA [50].

GRINA contains three potential ALG2-binding motifs (ABM1) that interact with the longest isoform of ALG2 (also known as PDCD6). This union is allosterically regulated by the conjugation of Ca^2+^ or Zn^2+^ to ALG2, which generates a hydrophobic binding pocket on ALG2. ALG2 senses Ca^2+^ and has been involved in the endosomal pathway, ER-stress-induced apoptosis, cell cycle progression, and cancer [43]. Interestingly, ALG2 is among the top RAR-related orphan receptor A (RORA)-linked genes with elevated expression in the hippocampus of patients with Alzheimer’s disease [44]. In response to a Ca^2+^ increase, ALG2 relocates to the vesicle coat protein complex II (COPII) that participates in anterograde vesicle transport from the ER to Golgi [45], whereas it translocates to the nucleus under heat shock stress or thapsigargin treatment [46], with help from the spliceosomal protein RBM22. It has been observed that all ABM1 motif-containing proteins enclose between 2 to 16 YP repeats that are recognized by a second hydrophobic pocket on the ALG2 surface [47]. GRINA includes 14 YP repeats. Moreover, all three ABM1 motifs are within a region homologous to the 33-mer gliadin peptide (Table 1, Figure 3). Intriguingly, alterations in the early-recycling endosomal system of cells derived from celiac patients increase gluten sensitivity [48], and, therefore, it is feasible that GRINA could be somehow altered.

The D^2^P^2^ software also reported the presence of MoRFs within the fragments 1–14, 66–105, and 114–122. MoRFs stand for Molecular Recognition Features and are IDRs that experience disorder-to-order transitions upon binding with their ligands.

The first MoRF comprises a BIR (baculoviral IAP repeat) sequence (MSHEK, position 1–5). BIR motifs are found in Inhibitor of apoptosis (IAP) proteins and bind specifically to IAP-binding motifs (IBMs) located at the N-termini of caspases and pro-apoptotic IAP-antagonizing proteins. Under normal conditions, IAPs bind to caspases suppressing their activity. However, upon apoptotic stimuli, pro-apoptotic IAP-antagonizing proteins, such as Smac, abrogate caspase inhibition by IAPs competing with them, which ultimately leads to cell death. As mentioned earlier, GRINA promotes cell survival through the modulation of ER Ca^2+^ homeostasis. Therefore, the presence of an IBM motif in a pro-survival protein like GRINA is surprising and deserves further research to confirm its role, if any. It has been suggested that competition among IBMs of different proteins may provide a mechanism to regulate pro-survival and cell death activities [49].

Interestingly, the second MoRF sequence vastly overlaps with the 63–96 fragment that shows homology with the 33-mer gliadin peptide [50]. Based on this homology (Figure 3), the 33-mer gliadin peptide may be able to interact with GRINA (establishing H bonds between the Pro present in the 33-mer and Gly residues of GRINA) and thus alter its functions. This biochemical mechanism would be relevant in many of the extraintestinal manifestations, such as schizophrenia, associated with patients who have celiac disease or non-celiac gluten sensitivity [50]. Indeed, about one third of the patients with schizophrenia harbor elevated inflammation and IgG antibodies against gliadin (anti-AGA). Supporting this association, a recent article [51] with 80 healthy controls and 160 patients with schizophrenia showed that the latter had increased gut permeability and higher levels of anti-GRINA antibodies compared to the controls, and that the presence of anti-GRINA antibodies was associated to anti-AGA antibodies. Despite the fact that they attributed their findings to molecular mimicry and cross-reactivity, it cannot be ruled out that the interaction among the 33-mer gliadin peptide and GRINA prompted the generation of anti-GRINA antibodies.

The Pro-rich sequence of GRINA also showed several non-specific hits that suggest other potential functions of GRINA in particular tissues. Among these, the CDD software uncovered homology with Atrophin-1 (E-value: 5.91 × 10^−9^), a protein found in neurons, which seems to function as a transcriptional co-repressor [52].

Another relevant case is the homology among the 42–142 residues of GRINA with the wheat glutenin of high molecular weight (HMW) (E-value: 3.37 × 10^−6^). It is worthy to note that HMW glutenins contain elastomeric motifs, and, together with gliadins, they contribute to the elastic properties of gluten and doughs. Moreover, an article by Rauscher et al. [53] revealed a threshold in Pro and Gly content, above which elastomeric properties appear and amyloid formation is hindered. The N-terminal tail of GRINA is above that threshold. Therefore, it is reasonable to think that the N-terminal Pro-rich domain of GRINA may confer it elastic properties, which would be helpful to bring together complex subunits or facilitate processes like endosome-to-Golgi retrieval, in which it participates [54].

IDRs could contribute with promiscuous interactions to promote the assembly of ribonucleoprotein granules [55] by the mechanisms previously noted. It is, therefore, possible that GRINA may exert a similar function at the nucleoli fibrillar centers, where it has been observed and ribosomes are synthesized and assembled, promoting survival under particular stress situations.

The GRINA Pro-rich region also shows homology to Bindin (E-value: 2.46 × 10^−5^), a protein involved in the species–specific adhesion of sperm to the egg surface during fertilization [56], which associates with phospholipid vesicles [57], and Gametogenetin (E-value: 9.99 × 10^−4^), a protein involved in the maturation of sperm that may take part in vesicular trafficking. Interestingly, GRINA has also been shown to be significantly expressed in testes [28,58], where it could exert similar functions.

## 5. The Subcellular Locations of GRINA

According to Nielsen et al. [10] GRINA is located in the Golgi apparatus of COS cells. This was confirmed by Lisak et al. [11] in HT22 cells. In addition, Breusegem and Seaman [54] also observed the protein in endosomes of HeLa cells, where it plays a role in the endosome-to-Golgi retrieval and its silencing results in partial loss of Golgi integrity.

Further, Rojas-Rivera et al. [28] identified GRINA in the ER of MEF cells, where it prevents apoptosis in response to intrinsic pathway stimuli and ER stress through the modulation of ER calcium homeostasis.

Finally, Hu et al. [59] and Mallmann et al. [29] found a significant expression of GRINA in the plasma membrane, where it is able to adjust the voltage-gated Ca_V_2.2 calcium channels in CHO cells.

### 5.1. The Unexplained Nuclear Staining of GRINA

However, none of these studies explain the staining observed in the nucleoli fibrillar centers of PC-3 and MCF7 cells and cytosol of PC-3 cells using the validated antibody HPA036981 (www.proteinatlas.org) [60]. This observation is counterintuitive, since GRINA has seven transmembrane segments that preclude its location outside membranes. The antibody recognizes the antigen sequence PYGQPQVFPGQDPDSPQHGNYQEEGPPSYYDNQDFPATNWDDKSIRQAFIRK (position 113–164) located in the N-terminal cytoplasmic tail (see Figure 1), and that includes several motifs conserved among the LFG family members (signature residues are underlined) [14,59].

### 5.2. The N-Terminal Half of GRINA Contains a Potential DNA-binding Sequence

For this reason, we investigated if there was any potential DNA-binding sequence within the N-terminal tail of GRINA using the DNABIND software [61]. Surprisingly, we found that the core sequence DKSIRQAFIRK (position 154–164) (Figure 1) reported the highest score and was considered a DNA-binding sequence with 100% probability, although longer sequences containing that fragment were also positive. This sequence is part of the antigen peptide recognized by the HPA036981 antibody.

### 5.3. The N-Terminal Half of GRINA May Be Cleaved or Alternatively Spliced to Enter the Nucleus

Therefore, it is feasible for whole GRINA to be synthesized under basal conditions and the cytoplasmic N-terminal half to be released from the rest under appropriate situations, like stress, thereby allowing it to remain soluble in the cytosol and eventually enter the nucleus. Otherwise, alternative splicing of GRINA may take place in such conditions and only express the exons that match with the N-terminal region, skipping the transmembrane domains. In either case, further processing may be possible by cellular proteases to generate a shorter fragment containing the DNA-binding sequence described above. Both scenarios are reasonable, since some proteins suffer alternative splicing under specific circumstances, while others can be released from their transmembrane counterparts after precise cleavage to act as transcription factors.

An interesting parallelism may be established with the ER stress sensor ATF6 (activating transcription factor 6), which traffics to the Golgi, where it is cleaved by S1P (site-1 protease) and S2P (site-2 protease), releasing a soluble fragment that translocates to the nucleus to regulate the transcription of genes involved in ER homeostasis [62]. Indeed, GRINA has been shown to interact with ATF6b in *Caenorhabditis elegans* [63].

Another relevant example involves the sterol regulatory element-binding proteins (SREBPs) coded by *SREBF1* and *SREBF2*, where SREBP1 is responsible for regulating de novo lipogenesis and SREBP2, the cholesterol metabolism. These transmembrane proteins located in the ER travel to the Golgi under low sterol levels (in the case of SREBP2) or in the presence of insulin and high acetyl-CoA levels (in case of SREBP1), where their cytosolic N-terminal fragments are released by the sequential action of the S1P and S2P transmembrane proteases, now able to enter the nucleus, with help from a karyopherin, and act as transcription factors, either promoting the synthesis of cholesterol or lipids. Eventually they are phosphorylated by GSK3 (glycogen synthase kinase 3), allowing their ubiquitinylation by the SCF (Skp1-Cul1-F-box-protein) ligase and consequent proteasomal degradation [64]. Again, GRINA has been shown to interact with SREBP1 in *C. elegans* [63,65].

Further supporting our hypothesis, we found cleavage sites for the S1P (also known as SKI1) (RQAF^I, position 158–162) and Nardilysin (also known as *N-*Arg dibasic convertase) (I^RK, position 162–164) proteases around the junction between the N-terminal region and the transmembrane half (see Figure 1 and Table 1). In addition, we detected possible phosphorylation sites within the N-terminal region that may be recognized by the GSK3, polo-like kinase (PLK)1, and PLK4 kinases [20]. It is worth noting that PLK4 locates at the SCF ubiquitin ligase complex. In particular, the sequence DDKSIRQ (position 153–159) is recognized by the cycle-dependent PLK1 (see Table 1), which reaches its maximal levels during the M phase and is important for regulating mitotic entry.

Although the N-terminal region does not encompass any characterized classical nuclear localization sequence (NLS) to import the peptide to the nucleus, it contains ten PY pairs that could be part of non-classical NLSs, known as PY-NLSs [66]. However, the described PY-NLSs are escorted so far by either basic or hydrophobic residues that are lacking in GRINA, and, consequently, their potential role in importing the cargo remains to be investigated.

However, additional evidence was discovered while searching the Conserved Domains Database (CDD) from the NCBI [21]. We found that the central part of the N-terminal region shows some homology with the provisional PHA03247 domain, represented by the large tegument protein UL36 of the human herpes simplex virus, also present in other *Herpesviridae*, such as the Epstein–Barr virus or cytomegalovirus. Interestingly, the C-terminus of pUL36 (with homology to GRINA) plays a role in routing the viral capsid to the nuclear pore complex [67], while the N-terminus (without homology to GRINA) stabilizes the substrates at the host nucleus by promoting the degradation of cullin-RING ubiquitin ligases [68].

That same N-terminal region of GRINA is also homologous to the ARC105/MED15 domain [21]. ARC105/MED15 is a subunit of the ARC/Mediator complex, a coactivator involved in the regulated transcription of most RNA polymerase II-dependent genes. A study on *C. elegans* [69] showed that worms lacking MDT-15, the homolog of MED15, activated their unfolded protein response (UPR) in the absence of disturbed proteostasis, but in reaction to altered ER membrane fluidity and composition. Hence, it is feasible that GRINA may sense such conditions in humans and get activated. Interestingly, a study published in *Nature* by Yang et al. [70] showed that MED15 is required by SREBP to activate target genes in the control of cholesterol and lipid homeostasis.

### 5.4. GRINA Affects Lipid and Cholesterol Metabolism

Therefore, it might be possible that GRINA, like SREBP, participates in lipid and cholesterol homeostasis under specific conditions. This possibility has been evidenced in a study with more than one thousand cows, were GRINA was among the enriched genes that significantly correlated (*p* < 0.008) to cholesterol concentration in milk [71], suggesting that GRINA might contribute to the regulation of cholesterol present in milk.

Since cholesterol affects membrane fluidity, enhances signal transport at synapses, and is protective against oxidative damage, it is not surprising that its metabolism is impaired in many neurodegenerative diseases, particularly knowing since the brain contains around 25% of the total cholesterol content (despite it only represents about 2.5% of the body weight), and over 95% of brain cholesterol is synthesized de novo by glial cells [72].

Further, *GRINA* was recently identified as one of the genes containing vitamin D receptor (VRD) super-enhancers [73]. Once vitamin D, which derives from cholesterol, is recognized by the VDR, the complex is able to enter the nucleus and bind to the super-enhancer DNA region present in *GRINA*, thereby exerting a higher regulatory effect on the expression of *GRINA* compared to typical enhancers and promoters. Moreover, the authors unveiled that for *GRINA* and other genes, these regions overlapped with Multiple Sclerosis risk single nucleotide polymorphisms [73]. Vitamin D is a natural ER stress reliever [74], supports protein homeostasis [75], inhibits adipogenesis [76], and also promotes alternative RNA splicing [77].

In addition, GRINA encloses two conserved motifs that can be recognized by the forkhead-associated (FHA) domain (Table 1), one of them right before the potential DNA-binding sequence. Proteins containing this domain have diverse cellular functions, such as vesicle transport, but this domain is particularly relevant in nuclear proteins involved in transcription regulation or cell cycle checkpoints. Their presence reinforces the idea that GRINA may participate in vesicle transport and the transcriptional regulation of cholesterol and lipids.

## 6. Transcriptional Regulation of GRINA

**Figure 4:**
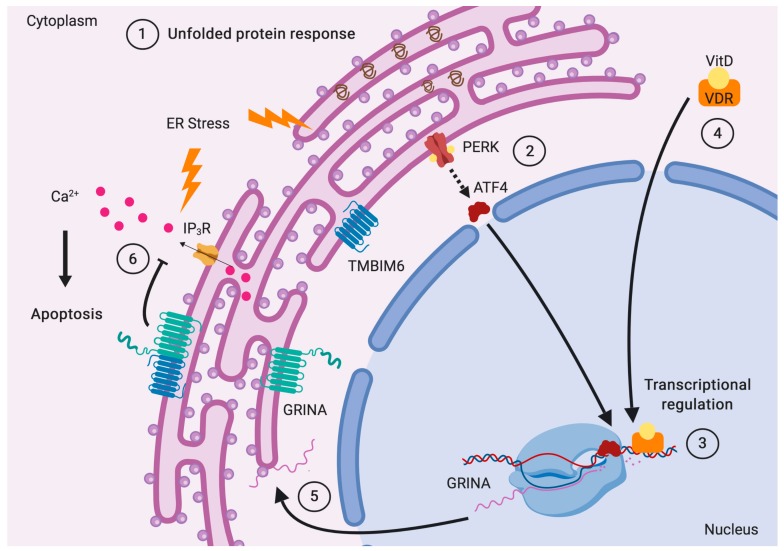
Transcriptional regulation of GRINA and its role in cell survival. (1) Upon ER stress the unfolded protein response is triggered. (2) The activated PERK arm allows the synthesis of the transcription factor ATF4, (3) which now can enter the nucleus and promote the transcription of GRINA. (4) Also, the vitamin D-VDR complex has been shown to enhance the expression of GRINA. (5) As a result, GRINA is synthesized (6) and able to interact with TMBIM6 to synergistically inhibit the release of Ca^2+^ by inositol triphosphate receptors (IP_3_Rs) towards the cytosol, thus suppressing the intrinsic apoptosis pathway.

The expression of GRINA is probably tightly regulated over time. Under ER stress, GRINA expression is upregulated by the PERK (protein kinase RNA-like endoplasmic reticulum kinase)/ATF4 pathway [28] (Figure 4). A study with NIH3T3 fibroblasts showed that GRINA mRNAs were among top 10% of mRNAs to undergo deadenylation [78], a process involved in gametogenesis, embryo development, cell cycle progression, and synaptic plasticity, which shortens mRNA poly(A) tails and plays an important role in mRNA stability and the translational efficiency of the target proteins.

A significant increase of the annotated translation initiation site of GRINA has been observed in response to KCl depolarization in murine neuron–glia cultures [79]. However, alternative promoter usage and significantly lower expression of GRINA was detected in postmortem superior temporal gyrus of schizophrenia patients in contrast to non-psychiatric controls [80,81].

The diversity of transcription factors (TF) that bind the promoter region of GRINA can illuminate the tissues and circumstances under which the expression of GRINA is relevant. To this purpose, a list of experimentally validated TFs that regulate the transcription of GRINA were obtained via the TF2 DNA database [82] and are presented below and in Table 2. Additional TFs can be found on the Harmonizome website [83] or be predicted with the TF2DNA [82].

- Aryl-hydrocarbon receptor nuclear translocator 2 (ARNT2) is almost exclusively expressed in the brain and prevents cell death [90].
- RORA participates in the induction of ER stress response [91]. It is a key factor in calcium homeostasis pathways and plays a role in spinocerebellar ataxias [92]. It governs postnatal cerebellar development [93] and may modulate antipsychotic response in schizophrenia [94]. RORA shows a circadian expression and senses fatty acids, cholesterol, and oxysterols [95]. It is upregulated in the hippocampus of patients with Alzheimer’s disease [44] and also regulates the expression of the tight junction protein CLDND1 in human brain endothelial cells [96].
- The cAMP responsive element binding protein 3-like 1 (*CREB3L1*) gene codes for the membrane-bound ER stress transducer OASIS, which has an important role in osteoblast differentiation during bone development [97]. OASIS promotes the expansion of the Golgi and the synthesis of transport factors to synchronize the rise in cargo load with the amplified capacity of the secretory pathway [98]. It is preferentially expressed in astrocytes in the central nervous system (CNS) [99] and modulated by the UPR to finely control astrocyte differentiation [100]. OASIS is also involved in the terminal differentiation of mucus-secreting goblet cells in the large intestine [101].
- PBX/knotted 1 homeobox 1 (PKNOX1) is essential during embryonic and postnatal development [102], such as in hindbrain development [103]. It contributes to mammary gland branching [104] and is required for adult spermatogenesis [102].
- Doublesex and mab-3 related transcription factor 1 (DMRT1) promotes spermatogenesis and prevents female reprogramming of male gonadal cells [105].
- Methyl-CpG binding domain protein 2 (MBD2) is upregulated at specific stages of spermatogenesis [106] and required for correct spatial gene expression in the gut [107]. Its loss protects mice against high-fat diet-induced obesity and insulin resistance [108].
- Inhibitor of DNA binding 4 (ID4) is up-regulated during embryogenesis and its highest expression in adult mice occurs in testes, brain and kidneys [109]. ID4 is required for the correct timing of neuronal differentiation [110] and essential for oligodendrogenesis [111]. Lack of ID4 drastically reduces osteoblast differentiation [112], but instead promotes de novo steroidogenesis [113].
- Estrogen-related receptor gamma (ESRRG) regulates testicular steroidogenesis through direct and indirect regulation of steroidogenic gene expression [114]. It positively regulates adipocyte differentiation [115] and modulates cell proliferation and estrogen signaling in breast cancer [116]. ESRRG also plays a key role in vascular calcification [117] and is involved in the determination of bone density in women [118]. It crosstalks with ATF6a to coordinate ER stress response [119] and is implicated in hearing loss and mild developmental delays [120]. ESSRG is highly expressed in the nervous system of mice embryos [121], and mice lacking neuronal ESSRG in the cerebral cortex and hippocampus exhibit defects in spatial learning and memory [122]. ESSRG is an essential coordinator of cardiac metabolism and function [123] and regulates cardiac, gastric, and renal K^+^ homeostasis [124].
- Myogenic factor 6 (MYF6) is involved in muscle differentiation, whereas HOXD8 is associated with the distal gut-associated mesoderm [125]. MESP1 is a key TF required for the induction of the cardiovascular gene expression program [126], and KLF13 regulates cardiac muscle development during the embryonic period [127] and mediates repression or activation under the control of SREBP-Sp1 [128].
- Zinc finger and BTB domain containing 7C (ZBTB7C) modulates DNA binding of SREBP-1c and Sp1 facilitating fatty acid synthesis [129].
- Histone H4 transcription factor (HINFP) is a co-activator in the sterol-regulated transcription of PCSK9, a target gene of SREBP2 that modulates the degradation of hepatic LDL receptors [130]. It is the only known TF required for the expression of histone H4 genes, and its loss compromises embryo implantation [131].
- ETS homologous factor (EHF) is epithelial-specific and regulates epithelial response to injury, including inflammation, efficient would repair, and barrier maintenance [132].
- Early growth response 3 (EGR3) regulates the expression of about 330 genes, 35% of which are involved in immune responses and inflammatory processes, and 15% crosstalk with the NF-κB signaling pathway [133]. It also regulates genes involved in synaptic plasticity, memory, and cognition [134] and has been associated with schizophrenia, bipolar disorder, and depression [135].
- Binding sites for NF-kB, which play a key role in inflammatory processes, are present also in the promoter region of adhesion molecules, cytokines, and growth factors [136].
- The *MYC* gene locates near GRINA at 8q24.21. Myc proto-oncogene (c-Myc) is a master regulator of oncogenesis, aerobic glucolysis [137], and immune response [138], coordinating protein synthesis through the transcriptional control of the ribosome’s biogenesis [139]. The expression of GRINA is upregulated in breast cancer, colorectal cancer and gastric cancer [13]. A recent article [87] confirmed that GRINA is upregulated in gastric cancer and that its regulation is mediated by c-Myc. The authors found that upon knockdown of GRINA, antiapoptotic Bcl-2 and Bcl-XL were downregulated, while proapoptotic Bax and Bak were upregulated. TMBIM6, TMBIM4, and TMBIM2 have also been shown to interact with the antiapoptotic proteins Bcl-2 and Bcl-XL [12]. Knockdown of GRINA also decreased CyclinD1 and CyclinE expression, which are involved in the G1 phase transition, and inhibited phosphorylation of Akt and the downstream effector mTOR, indicating that GRINA modulates aerobic glycolysis though that pathway.

## 7. The Draft Protein–Protein Interaction Network of GRINA

Available information about the protein–protein interaction network of GRINA is very limited and can be found online at the Human Reference Protein Interactome mapping project (interactome.baderlab.org), String v11.0 [140], or FunCoup v4.0 [141] websites. In order to complete this information, here we introduce our preliminary results. The full list of protein ligands can be found in Table S1, together with the materials and methods, and is briefly discussed below.

### 7.1. Ticket to the Nucleus

**Figure 5:**
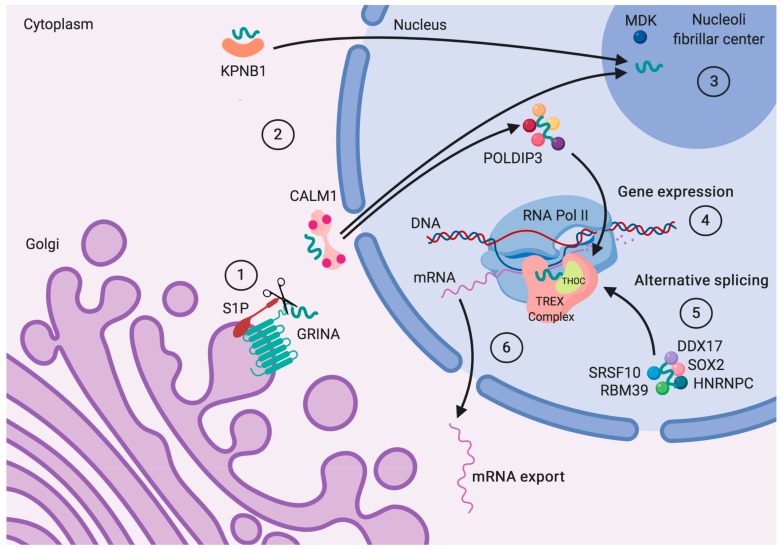
A model for the nuclear localization of GRINA. (1) Under appropriate conditions the N-terminal side of GRINA may be cleaved by S1P or Nardilysin. (2) Then, it may be able to enter the nucleus assisted either by Karyopherin β1 (KPNB1) (under resting conditions) or CALM1 (under stress). Once there, GRINA may play several roles. (3) It may enter the nucleoli fibrillar centers to facilitate the transcription of ribosomal RNAs in collaboration with MDK. (4) It also may bring together components of the TREX complex, such as POLDIP3, to facilitate mRNA stability, (5) and associate with other transcription factors like SOX2, and proteins involved in alternative splicing (DDX17, SRSF10, RBM39, HNRNPC) to promote the transcription and (6) mRNA export of the right subset of genes under the new changing conditions.

We found that GRINA is able to interact with Karyopherin β1 (KPNB1) and Calmodulin (CaM), which reinforces our hypothesis that the N-terminal half of GRINA may be able to enter the nucleus, using KPNB1 under resting conditions, and CaM under stress conditions (see Figure 5).

Karyopherins are responsible of the nuclear import of proteins and can be classified in two subfamilies (α and β). Depending on the type and isoform, karyopherins recognize a particular NLS present in the cargo protein. Whereas some Karyopherin βs recognize their cargo directly via NLSs, others require Karyopherin α adaptors [142]. Only some NLSs have been characterized. For Karyopherin β2, the consensus NLS has either a hydrophobic or basic motif followed by [RHK]X{2–5}PY [66]. Upon stress, several karyopherins, like Karyopherin β1, stop shuttling between the cytoplasm and the nucleus and remain hijacked in cytoplasmic stress granules [143].

Then, another pathway dependent on Ca^2+^, rather than GTP, is triggered. The increase of cytoplasmic Ca^2+^ activates the calmodulin-dependent import pathway, which recognizes and imports a specific subset of NLS-bearing proteins [144]. In addition to that, CaM (which is encoded by the non-allelic genes Calmodulin 1 (*CALM1*), *CALM2*, and *CALM3*, which produce an identical protein) is known to be involved in the Ca^2+^-dependent inactivation of several types of voltage-gated Ca^2+^ channels [145,146], including the Ca_V_2.2 channels [147] (Figure 2). Ca_V_ channels have a major effect on cellular Ca^2+^ influx and increase intracellular Ca^2+^ concentration. In particular, high voltage-activated N-type Ca_V_2.2 channels are predominantly expressed at the presynaptic neuronal terminals where they trigger a fast release of neurotransmitters through their interaction with the SNARE complex [148]. Ca_V_2.2 channels only undergo Ca^2+^-dependent inactivation but not facilitation, which is due to their inability to transduce the effects of CaM, rather than weak binding to CaM, per se [149]. CALM1 also participates in cytoskeleton remodeling and is essential for the migration of mouse precerebellar neurons [150].

### 7.2. Role in Vesicle Traffic and Cell Adhesion

Other groups of proteins were related to the cytoskeleton, motility, and vesicular trafficking, such as members of the microtubules (TUBA1C), microfilaments (ACTG1, CAPZB), and the proteins that interact with them (MYO1B, JUP). Myosin 1B (MYO1B) is a ubiquitous cellular protein that promotes axon formation in neurons [151] and modulates the cargo sorting within multi-vesicular endosomes and their morphology [152] (Figure 2). Reinforcing our results, GRINA has been previously reported to be required for endosome-to-Golgi retrieval [54]. According to this result, the loss of GRINA alters the levels of endosome-to-Golgi cargo proteins, and fragmentation of *cis*-Golgi (without affecting the retromer integrity) is observed when GRINA is silenced. On the other hand, transfection of GRINA provokes a decrease of the *trans*-Golgi network but an increase of the endosomes [54]. Deregulation of retromer-mediated endosomal protein sorting leads to various pathologies, including Alzheimer’s disease.

Plakoglobin (JUP) is a γ-catenin and a component of the desmosomes and zonula adherens that occur at cell–cell junctions. As mentioned before, the KE (position 370–371) and SPEEY (position 340–344) motifs have been shown to be important for cell adhesion, as well as the motifs at the N-terminal half recognized by SH3 domains (see Table 1).

### 7.3. Alternative Splicing to Face Changing Conditions

We also detected several nuclear proteins, most of them involved in alternative splicing. This mechanism increases the diversity of proteins produced by a single gene, allowing the cell to adjust better to particular conditions, like ER stress (see Figure 5).

Heterogeneous nuclear ribonucleoprotein C (hnRNPC) is an abundant RNA-binding protein responsible for pre-mRNA splicing and may be involved in the fine-tuning of vitamin D-regulated target gene expression [77].

Pre-mRNA processing factor 3 (PRPF3) associates with U4 and U6 RNPs and has a role in pre-mRNA splicing. Mutations in this gene cause retinitis pigmentosa [153].

DEAD-box helicase 17 (DDX17) is an RNA helicase involved in spliceosome, ribosome assembly, and translation initiation. It may also have a role in retinitis pigmentosa and regulates alternative splicing in conditions like cell differentiation [154] and migration [155]. Moreover, it acts as a master regulator of steroid hormone-signaling pathways by controlling the transcription and splicing, both upstream and downstream, of estrogen- and androgen-receptors [156].

Sex determining region Y-box 2 (SOX2) is a critical transcription factor for early embryogenesis that is required for stem-cell maintenance in the CNS. SOX2 also regulates gene expression in the stomach. Curiously, SOX2 physically interacts with several hnRNPs, including hnRNPC [157] and DDX17 [158], suggesting an additional role in post-transcriptional regulation.

RBM39 is a splicing factor that acts as a transcriptional coactivator for the steroid nuclear receptors JUN, ESR1, and ESR2 [159]. RBM39 is a PUF60 paralog, another splicing factor that has been shown to interact with GRINA in *Drosophila melanogaster* [160].

Polymerase delta-interacting protein 3 (POLDIP3) interacts and activates the DNA polymerase δ [161] and is involved in the regulation of the translation of IFN-α-related mRNAs [162] and cell growth [163]. It belongs to the transcription-export (TREX) complex [164] that is responsible for mRNA stability and nuclear export. This process is critical for synapse development and alteration in POLDIP3 splicing is associated with amyotrophic lateral sclerosis [165].

SRSF10 participates in constitutive and regulated RNA splicing, having different effects depending on its phosphorylation in response to heat shock [166] or the isoform used in neurons [167]. HMGA1 is a key nonhistone protein involved in chromatin remodeling and is overexpressed in cancer [168].

Midkine (MDK) is a growth factor that is highly expressed in the mid-gestational period and induced in reactive astrocytes by ischemia insults [169]. It mediates neurite outgrowth and osteoblast cell migration [170]. It can be internalized by the low-density lipoprotein (LDL) receptor-related protein (LRP) and further transported to the nuclei depending on nucleolin [171]. MDK accumulates in the nucleolus (mainly at the granular component, dense fibrillar component and the border with the fibrillar component), where GRINA has been also localized, and is involved in rRNA transcription for ribosome biogenesis [171,172]. The nuclear targeting of MDK is also related to increased cell survival and anti-apoptotic activity [173].

IDRs present in GRINA can serve as scaffolding, accelerating the interactions between the binding partners by raising their local concentrations [34]. This may be the case for many of the ligands detected for GRINA—such components of alternative splicing, the TREX complex, the cytoskeleton, the nucleolus, or the ribosome. Indeed, MoRF-containing proteins, like GRINA, tend to interact among them and are enriched in the ribosome, nucleus, nucleolus, and microtubules, and are involved in translation, protein transport, protein folding, and interactions with DNA [174].

## 8. Conclusions

GRINA contains two main domains that confer it the necessary properties for its functions. The LFG domain spans the C-terminal half coinciding with the transmembrane region, whereas the Pro-rich domain comprises the cytoplasmic N-terminal half. GRINA can regulate Ca^2+^ homeostasis by either acting as a channel itself (with several residues from the LFG domain involved) or modulating other proteins like Ca_V_2.2 or IP_3_Rs, alone or in combination with other members, such as TMBIM6 or CaM. Ca^2+^ regulation is vital to promote cell survival under ER stress conditions but also in more specialized situations, such as calcification in bones or neurotransmission in the brain. GRINA is also involved in vesicle transport and sorting, as exemplified by Gb3 synthase, and it can move between the ER and other membrane compartments (Golgi, endosome, and cell surface), likely mediated by ALG2, MYOB, and the di-Arg motif exposure. All these processes, including endosome-to Golgi retrieval, are especially relevant in secretory cells like neurons, mammary glands, or the digestive tract. To conclude, the N-terminal cytoplasmic fraction of GRINA contains a potential DNA-binding sequence that may be synthesized by alternative splicing or released from the whole protein by the S1P or Nardilysin proteases, allowing it to enter the nucleus with assistance of KPNB1 or CaM, where it may regulate the transcription, alternative splicing and mRNA export of a subset of genes involved in lipid and cholesterol synthesis, ribosome biogenesis, or cell cycle progression.

## Figures and Tables

**Table 1:** List of conserved linear motifs in GRINA from the eukaryotic linear motif (ELM) resource [20].

ELM Name	Motif	Position	C.S.^1^	Pattern & Description	Cell Compartment
CLV_NRD_NRD_1	IRK RRK	162–164 221–223	1.0 0.98	(.RK)|(RR[^KR]) *N*-Arg dibasic convertase (NRD/Nardilysin) cleavage site (X-|-R-K or R-|-R-X)	extracellular, Golgi, cell surface
CLV_PCSK_SKI1_1	RQAFI	158–162	1.0	[RK].[AILMFV][LTKF]. Subtilisin/kexin isozyme-1 (SKI1/S1P) cleavage site ([RK]-X-[hydrophobic]-[LTKF]-|-X)	ER, Golgi, extracellular
LIG_BIR_II_1	MSHEK	1–5	1.0	^M{0,1}[AS]... Inhibitor of Apoptosis (IAP)-binding motifs (IBM) are found in pro-apoptotic proteins and bind to type II BIR domains present in IAPs	cytosol, mitochondrion
LIG_EF_ALG2_ABM_1	PYPQGGYP PYPQGGYP PYPQGGYP	63–70 73–80 93–100	0.85 0.85 0.85	P[PG]{0,1}YP.{1,6}Y[QS]{0,1}P This isoform-specific ALG2-binding motif binds to the EF hand domains of the proapoptotic Ca^2+^-binding ALG2 protein in a Ca^2+^-dependent manner	nucleus, cytosol, ESCRT I complex, endosome, ER
LIG_SH3_3	NYPPPNP PPNPGYP GPQPPMP YAQPPYP EGYPQGP	13–19 16–22 24–30 32–38 87–93	0.73 0.73 0.5 0.77 0.82	...[PV]..P This is the motif recognized by those SH3 domains with a non-canonical class I recognition specificity	plasma membrane, focal adhesion, cytosol
LIG_FHA_2	PATNWDD MQTRYDF	148–154 273–279	0.72 1.0	..(T)..[DE]. Phospho-Thr motif binding a subset of FHA domains that have a preference for an acidic amino acid at the pT+3 position	nucleus, replication fork
LIG_TRAF2_1	SPEE	340–343	1.0	[PSAT].[QE]E Major TRAF2-binding consensus motif. Members of the tumor necrosis factor receptor (TNFR) superfamily initiate intracellular signaling by recruiting the C-domain of the TNFR-associated factors (TRAFs) through their cytoplasmic tails	cytosol
DOC_WW_Pin1_4	LSLSPE	337–342	1.0	...([ST])P. The Class IV WW domain interaction motif is recognised primarily by the Pin1 phosphorylation-dependent prolyl isomerase	nucleus, cytosol
MOD_CK2_1	LSLSPEE	337–343	1.0	...([ST])..E CK2 phosphorylation site	nucleus, cytosol
MOD_ProDKin_1	LSLSPEE	337–343	1.0	...([ST])P.. Pro-Directed Kinase (e.g., MAPK) phosphorylation site in higher eukaryotes	nucleus, cytosol
MOD_Plk_1	DDKSIRQ	153–159	0.96	.[DNE][^PG][ST](([FYILMVW]..)|([^PEDGKN][FWYLIVM]).) Ser/Thr residue phosphorylated by the Plk1 kinase	nucleus, spindle, γ-tubulin complex, midbody, cytosol, kinetochore, nuclear condensin complex, cleavage furrow, nucleoplasm, microtubule organizing center
TRG_ER_diArg_1	FRR	220–222	1.0	([LIVMFYWPR]R[^YFWDE]{0,1}R)|(R[^YFWDE]{0,1}R[LIVMFYWPR]) The di-Arg ER retention motif is defined by two consecutive arginine residues (RR) or with a single residue insertion (RXR). The motif is completed by an adjacent hydrophobic/Arg residue which may be on either side of the Arg pair	cytosol, ER, ER-Golgi transport vesicle

^1^ Conservation Score.

**Table 2:** Experimentally validated transcription factors that regulate the transcription of human GRINA by the RNA polymerase II (value: 0.0001), according to the TF2DNA database [82].

Classification	TF	Description	References
Basic domain	ARNT2	Aryl-hydrocarbon receptor nuclear translocator 2	[84]
	MYF6	Myogenic factor 6 (Herculin)	[84]
	ID4	Inhibitor of DNA binding 4, dominant negative helix-loop-helix protein	[85]
	CREB3L1	cAMP responsive element binding protein 3-like 1	[85]
	MESP1	Mesoderm posterior bHLH transcription factor 1	[85]
	c-MYC	Myc proto-oncogene	[86,87]
Immunoglobulin fold	NFκB1	Nuclear factor of kappa light polypeptide gene enhancer in B-cells 1	[85,88]
Helix-turn-helix domain	HOXD8	Homeobox D8	[85]
	EHF	ETS homologous factor	[84]
	PKNOX1	PBX/knotted 1 homeobox 1	[84]
Zinc-coordinating DNA-binding domain	DMRT1	Doublesex and mab-3 related transcription factor 1	[89]
	ZBTB6	Zinc finger and BTB domain containing 6	[88]
	ZBTB7C	Zinc finger and BTB domain containing 7C	[85]
	ESRRG	Estrogen-related receptor gamma	[84]
	MBD2	Methyl-CpG binding domain protein 2	[84]
	RORA	RAR-related orphan receptor A	[84]
	EGR3	Early growth response 3	[85]
	HINFP	Histone H4 transcription factor	[85]
	KLF13	Kruppel-like factor 13	[85]

## Supplementary Materials

Supplementary materials can be found at https://www.mdpi.com/1422-0067/20/16/4005/s1.

## Abbreviations

LFG	Lifeguard
IDR	Intrinsically Disordered Region
MoRF	Molecular Recognition Feature
ER	Endoplasmic Reticulum
SREBP	Sterol Regulatory Element-Binding Element
LDL	Low-Density Lipoprotein
ABM1	ALG2-Binding Motif 1
IBM	IAP-Binding Motif
NLS	Nuclear Location Sequence
TF	Transcription Factor
