# Supplementary material for: Deciphering GRINA/Lifeguard1: Nuclear Location, Ca2+ Homeostasis and Vesicle Transport
Source: Int J Mol Sci. 2019 Aug 16;20(16):4005. doi: 10.3390/ijms20164005 (PMC6719933; doi:10.3390/ijms20164005)
Supplement: Supplementary file 1 [file ijms-20-04005-s001.pdf]

International Journal of *Molecular Sciences*

MDPI

Supplementary data

Deciphering GRINA/Lifeguard1: nuclear location, Ca^{2+} homeostasis and vesicle transport

Víctor Jiménez-González, Elena Ogalla-García, Meritxell García-Quintanilla and Albert García-Quintanilla

Supplementary Table 1. Draft protein-protein interactions of human GRINA.

Uniprot	Description	Gene
Q9BY77-2	Polymerase delta-interacting protein 3	POLDIP3
Q59F66	DEAD box polypeptide 17 isoform p82 variant	DDX17
Q59GA1	Splicing factor, arginine/serine-rich 10 (Transformer 2 homolog, Drosophila) variant	SRSF10
B4DRA0	cDNA FLJ58459, highly similar to RNA-binding region-containing protein 2	RBM39
D3DPI2	HCG1641229, isoform CRA_a	HNRNPC
B4DLS8	cDNA FLJ55983, highly similar to U4/U6 small nuclear ribonucleoprotein Prp3	PRPF3
P17096-2	High mobility group protein HMG-I/HMG-Y	HMGA1
B2RBR9	cDNA, FLJ95650, highly similar to Homo sapiens karyopherin (importin) beta 1 (KPNB1), mRNA	KPNB1
B2R6J0	cDNA, FLJ92971, highly similar to Homo sapiens SRY (sex determining region Y)-box 2 (SOX2), mRNA	SOX2
B4DE59	cDNA FLJ60424, highly similar to Junction plakoglobin	JUP
A8K1M2	cDNA FLJ75174, highly similar to Homo sapiens calmodulin 1 (phosphorylase kinase, delta), mRNA	CALM1
O43795-2	Unconventional myosin-Ib	MYO1B
B4E3A4	cDNA FLJ57283, highly similar to Actin, cytoplasmic 2	ACTG1
B4DWA6	cDNA FLJ60094, highly similar to F-actin capping protein subunit beta	CAPZB
E9PGK3	Tubulin alpha 1c	TUBA1C
E9PPJ5	Midkine	MDK
Q5HY50	60S ribosomal protein L10	RPL10
B7Z4K2	60S ribosomal protein L31	RPL31
C9JEE0	Immunoglobulin lambda-like polypeptide 1	IGLL1
P01594	Immunoglobulin kappa variable 1-33	IGKV1-33
B0UZ85	Complement C4B (Chido Blood Group)	C4B

The interaction between GRINA and some of the ribosomal components could assist ribosome assembly, however upon ER stress, a massive detachment of translating ribosomes from the ER membrane takes place [1]. Therefore, it opens the appealing possibility that ribosomal detachment could be facilitated by GRINA as a result of its interaction with L10 and L31 proteins of the 60S subunit, which would provoke a conformational change of the large subunit [2], stopping translation and eventually avoiding the accumulation of unfolded proteins.

IGLL1 and IGKV1-33 are probably contaminants due to the antibody used for capturing GRINA. The complement component C4 is mainly found in the blood and produced by hepatocytes, therefore it should be considered a contaminant present in the serum of the cell cultures. However, immune cells can also contribute to the production of members of the complement system [3], and therefore it could have been produced by the HAP1 cells, which are derived from chronic myelogenous leukemia.

Our preliminary list of candidates included other members of the TREX complex, in addition to POLDIP3, that were excluded for being part of the Peptracker list. These proteins and their frequency of detection were THOC1 (22.2%), THOC2 (28.4%), THOC5 (24.4%) and ZC3H11A (29.3%). Interestingly, TMBIM4 has been identified as a THOC5 target gene [4].

We also obtained MYO1F and KIFC1, that are known to participate in endosome-to-Golgi retrieval [5], but they were discarded for being part of the Peptracker list with a detection frequency of 15.6% and 23.9%, respectively.

Despite the valuable information obtained by mass spectrometry, this technique has several limitations to study GRINA. First, any cell line used is limited in the profile of proteins it expresses. Also, the relative abundance of the fragments generated is critical and they can mask less abundant peptides, providing false negatives. In addition, IDRs such as those present in GRINA, are Pro-rich sequences that usually lack cleavage sites for enzymatic digestion, which hampers the generation of unique fragments or with the optimal size to be detected. Moreover, IDRs tend to form weak and promiscuous interactions, which may contribute to omit real ligands and include others generally regarded as contaminants.

Materials and Methods

To study the protein-protein interactions of GRINA, we acquired the parental HAP1 cell line expressing GRINA and the corresponding mutant, knock-out (KO) for GRINA, generated by CRISPR-Cas9 technology (Horizon Discovery) as negative control. The cells were handled in sterile biosafety cabinets at the General Service of Biology (CITIUS, University of Seville) and cultured in IMDM media with 10% fetal calf serum plus Penicilin (100 U/mL) and Streptomycin (100 μg/mL) at 37 ºC and 5% CO_2 atmosphere. Immunoprecipitation of total proteins was performed with the Capturem IP & Co-IP kit (Clontech) following the manufacturer's instructions. For the capture of GRINA we used the rabbit polyclonal antibody AP13558c (Abgent) that was raised against the antigenic region 131-158 of the N-terminal tail, at a 1:40 dilution. The immunoprecipitation procedure allows the discovery of both direct and indirect interactions as well as those shaped by multiple ligands. The eluates were sent to the Service of Proteomics (Institute of Biomedicine of Seville, Seville) where the samples were digested with Lys-C and Trypsin, and analyzed with the Q Exactive plus hybrid quadropole-Orbitrap and nano-HPLC Easy n-LC 1000 integrated system (Thermo Fisher Scientific). Peptides present in the KO sample as well as frequent contaminants listed in the Peptracker list [6] were discarded from the results, ensuing 21 potential ligands for GRINA that are shown in Supplementary Table 1 above. General information about the candidates was retrieved from Genecards [7].

References

1. Reid, D.W.; Chen, Q.; Tay, A.S.-L.; Shenolikar, S.; Nicchitta, C. V. The Unfolded Protein Response Triggers Selective mRNA Release from the Endoplasmic Reticulum. *Cell* **2014**, *158*, 1362–1374.
2. Potter, M.D.; Seiser, R.M.; Nicchitta, C. V. Ribosome exchange revisited: A mechanism for translation-coupled ribosome detachment from the ER membrane. *Trends Cell Biol.* **2001**, *11*, 112–115.
3. Lubbers, R.; van Essen, M.F.; van Kooten, C.; Trouw, L.A. Production of complement components by cells of the immune system. *Clin. Exp. Immunol.* **2017**, *188*, 183–194.
4. Saran, S.; Tran, D.D.H.; Ewald, F.; Koch, A.; Hoffmann, A.; Koch, M.; Nashan, B.; Tamura, T. Depletion of three combined THOC5 mRNA export protein target genes synergistically induces human

hepatocellular carcinoma cell death. *Oncogene* **2016**, 35, 3872–3879.

5. Breusegem, S.Y.; Seaman, M.N.J. Genome-wide RNAi Screen Reveals a Role for Multipass Membrane Proteins in Endosome-to-Golgi Retrieval. *Cell Rep.* **2014**, *9*, 1931–1945.
6. Boulon, S.; Ahmad, Y.; Trinkle-Mulcahy, L.; Verheggen, C.; Cobley, A.; Gregor, P.; Bertrand, E.; Whitehorn, M.; Lamond, A.I. Establishment of a Protein Frequency Library and Its Application in the Reliable Identification of Specific Protein Interaction Partners. *Mol. Cell. Proteomics* **2010**, *9*, 861–879.
7. Stelzer, G.; Rosen, N.; Plaschkes, I.; Zimmerman, S.; Twik, M.; Fishilevich, S.; Iny Stein, T.; Nudel, R.; Lieder, I.; Mazor, Y.; et al. The GeneCards suite: From gene data mining to disease genome sequence analyses. *Curr. Protoc. Bioinforma.* **2016**, *54*, 1.30.1-1.30.33.

© 2019 by the authors. Submitted for possible open access publication under the terms and conditions of the Creative Commons Attribution (CC BY) license (http://creativecommons.org/licenses/by/4.0/).
